# Measuring implanted patient response to tone pips

**DOI:** 10.1186/s12938-020-00844-6

**Published:** 2021-01-14

**Authors:** Juan M. Cornejo, Agar K. Quintana, Nohra E. Beltran, Pilar Granados

**Affiliations:** 1 Electrical Engineering Department, Biomedical Engineering Area, Metropolitan Autonomous University, Av. San Rafael Atlixco 186, Leyes de Reforma 1ra Secc, 09340 Iztapalapa, CDMX Mexico; 2 Gratuate Program in Biomedical Engineering, Metropolitan Autonomous University, Av. San Rafael Atlixco 186, Leyes de Reforma 1Ra Secc, 09340 Iztapalapa, CDMX Mexico; 3 Process and Technology Department, Metropolitan Autonomous University, Vasco de Quiroga 4871, 05348 Cuajimalpa de Morelos, CDMX Mexico

**Keywords:** Pediatric population, Current dynamic range, Objective test, Cochlear implant fitting

## Abstract

**Background:**

An electrical potential not previously reported—electrical cochlear response (ECR)—observed only in implanted patients is described. Its amplitude and growth slope are a measurement of the stimulation achieved by a tone pip on the auditory nerve. The stimulation and recording system constructed for this purpose, the features of this potential obtained in a group of 43 children, and its possible clinical use are described. The ECR is obtained by averaging the EEG epochs acquired each time the cochlear implant (CI) processes a tone pip of known frequency and intensity when the patient is sleeping and using the CI in everyday mode. The ECR is sensitive to tone pip intensity level, microphone sensitivity, sound processor gain, dynamic range of electrical current, and responsiveness to electrical current of the auditory nerve portion involved with the electrode under test. It allows individual evaluation of intracochlear electrodes by choosing, one at the time, the central frequency of the electrode as the test tone pip frequency, so the ECR measurement due to a variable intensity tone pip allows to establish the suitability of the dynamic range of the electrode current.

**Results:**

There is a difference in ECR measurements when patients are grouped based on their auditory behavior. The ECR slope and amplitude for the Sensitive group is 0.2 μV/dB_HL_ and 10 μV at 50 dB_HL_ compared with 0.04 μV/dB_HL_ and 3 μV at 50dB_HL_ for the Inconsistent group. The clinical cases show that adjusting the dynamic range of current based on the ECR improved the patient’s auditory behavior.

**Conclusions:**

ECR can be recorded regardless of the artifact due to the electromyographic activity of the patient and the functioning of the CI. Its amplitude and growth slope versus the intensity of the stimulus differs between electrodes. The relationship between minimum ECR detection intensity level and auditory threshold suggests the possibility of estimating patient auditory thresholds this way. ECR does not depend on the subject’s age, cooperation, or health status. It can be obtained at any time after implant surgery and the test procedure is the same regardless of device manufacturer.

## Background

Objective measurements such as electrical auditory brainstem response (eABR), electrically evoked stapedial reflex threshold (eSRT), and electrically evoked compound action potential (eCAP) provide alternatives to the behavioral CI fitting method in the pediatric population [1–3]. In these measurements biphasic current pulses are used as test stimulation, sent in monopolar mode to individually selected intracochlear electrodes through an interface unit provided by the device manufacturer. Except the eCAP, the electrical stimulating pulses are similar to those used in programming the CI, obtaining the auditory system response as a change of immittance in the ear contralateral to the CI, as in the eSRT case, or as a single-channel electrical recording in differential mode using surface electrodes, in the case of the eABR.

These objective measurements help to predict the limit values of the dynamic range of stimulating electrical current, which, applied through intracochlear electrodes, ideally gives the patient useful, comfortable, and safe hearing. This is achieved by matching the minimum and maximum levels of the dynamic range of electrical current to the *T* level—psychophysical auditory threshold—and the *C* or M/C level—psychophysical auditory comfort level or maximum auditory comfort level—, in each of the intracochlear electrodes [2, 4, 5].

Some reasons which limit the CI user’s ability to detect and discriminate sounds are the health status of the implanted cochlea, which results in variations of sensitivity to electrical current depending on the region stimulated, in addition to the parameters used to configure the stimulating electrical current [6–10].

Today the objective method most widely used for CI fitting is the eCAP threshold measuring, which in turn is used to predict the minimum and maximum values of the dynamic range of stimulating electrical current in one or more intracochlear electrodes. In addition, these values are used to establish the dynamic range of stimulating electrical current in the remaining electrodes. However, both the electrical current parameters and the configuration of the stimulating electrodes used to obtain the eCAP differ from those used in everyday operation of the device. It is noteworthy that it is not necessary to consider the operation of the CI sound processor in this methodology [1, 11–13].

The time restrictions imposed by the refractory period of the auditory nerve and the noise cancelation method used to diminish the electrical artifact of the test pulse to obtain the eCAP cause test pulse width values higher than 25 µs, and test current stimulation rate values below 500 Hz, to be different from those used in everyday operation of the CI, unlike the level of the test current, which may reach suprathreshold values.

For a certain input sound, the loudness experienced by the CI user will depend on the level of electrical current the CI assigns to the intracochlear electrodes and the auditory nerve responsiveness to electrical current. The correspondence between the input sound intensity level and the level of electrical current assigned to each intracochlear electrode depends on microphone sensitivity, sound processor gain, gain of the bandpass filter of each electrode, pulse width, and dynamic range of electrical current for each intracochlear electrode.

The importance of determining the dynamic range of electrical current lies in that the sound intensity of the different spectrum bands in which the CI divides input sound is translated into a level of current assigned to each intracochlear electrode, which is chosen from within the interval of values delimited by the dynamic range of electrical current of the corresponding intracochlear electrode [14].

The methodological considerations used to obtain the optimum eCAP threshold level for use in determining the dynamic range of current in intracochlear electrodes do not weight the relationship of patient auditory thresholds with rate of stimulation, pulse width, and effects of integration to auditory nerve response from adjacent pulses equally apart when stimulating with pulse trains of variable amplitude and rates of stimulation above 1000 pps [15, 16], a scenario which corresponds more closely to the everyday usage conditions of the CI.

To date, the success achieved in matching the extreme values of the dynamic range of electrical current to patients’ psychophysical levels T and C/M using any of the currently available objective tests has been limited. Therefore, to know the result of CI fitting or changes in levels of stimulation, it is necessary to observe the development of patient auditory behavior over time.

A different way of approaching this problem is to have a measurement of auditory nerve response in the implanted patient due to an input sound. This is possible by recording the scalp electrical potential generated when an intracochlear electrode stimulates the patient’s auditory nerve, every time CI processes an external tone pip of known intensity and frequency.

Such is the case of the electrical potential we have called electrical cochlear response (ECR), which is observed only in implanted patients. It is generated when an electrical current is established between an intracochlear electrode—active or stimulating electrode—and an extracochlear electrode—passive or reference electrode—of sufficient level to trigger an electrical response from auditory nerve in proximity to the stimulating electrode and in the pathway the electrical current follows between this pair of electrodes. On the contrary, ECR will not be generated if the electrical current flowing between these two electrodes is not of a level sufficient to excite the auditory nerve [17].

In general, the tissue, intracochlear and extracochlear, located between the active and passive electrodes, excitable or not, opposes the passage of electrical current. This opposition—or impedance—to the passage of electrical energy depends, in addition to anatomical and physiological factors, on the rate of stimulation (frequency, *ω*) of biphasic electrical current. In this context, impedance* z*(*jω*) can be understood as the quotient of the electrical current $i\left(jw\right)$ which flows between the pair of electrodes and the voltage $v(jw)$ measured at scalp.

According to Ohm’s law $\left|v(jw)\right|=\left|z(jw)\right|\cdot \left|i(jw)\right|$, i.e., the magnitude of the voltage $\left|v(jw)\right|$—ECR amplitude—is equal to the magnitude of impedance between the active and reference electrodes multiplied by the magnitude of the electrical current. When the active and reference electrodes are chosen, the impedance magnitude, $\left|z(jw)\right|$, can be considered constant. Thus, variations in current magnitude, $\left|i(jw)\right|$, will manifest as changes of magnitude of voltage, $\left|v\left(jw\right)\right|.$ This simple relationship allows us to consider ECR amplitude as a measurement of the stimulation experienced by the auditory nerve in response to a test tone pip of known frequency and intensity.

**Fig. 1:**
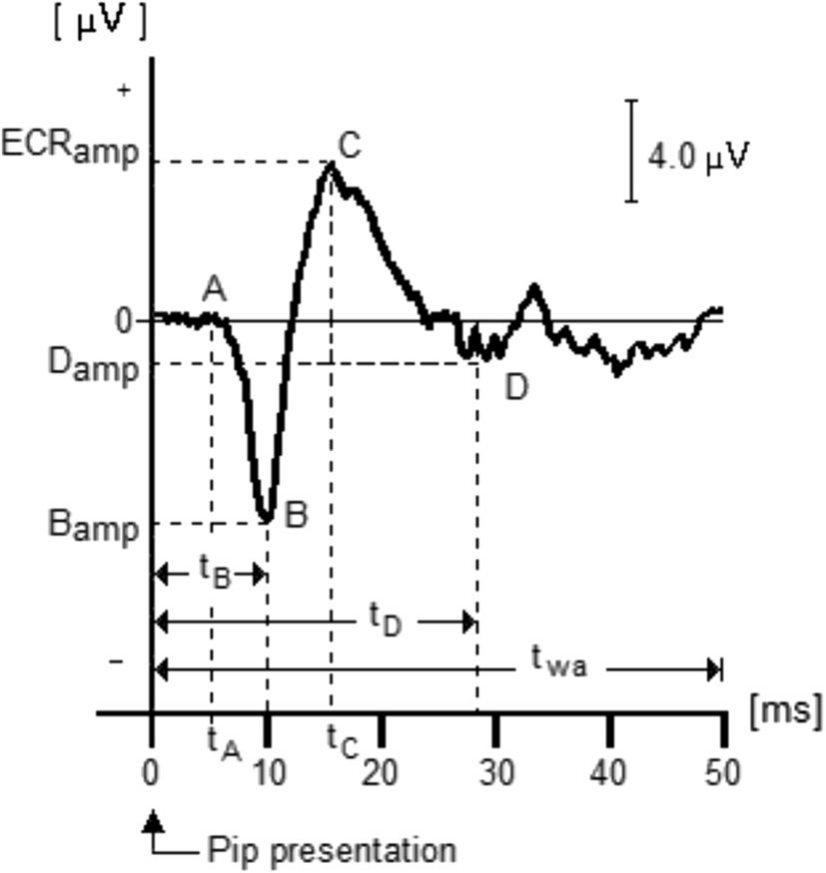
Typical electrical cochlear response (ECR) morphology. It is generated due to electrical stimulation delivered to the patient’s auditory nerve each time the CI process a tone pip of known frequency and intensity. The figure illustrates the ECR due to a 20 ms, 1518 Hz at 60 dB_HL_ tone pip presented in sound field with patient using the device in everyday mode and positioned 0.60 m away from the sound source

Typical ECR morphology is shown in Fig. 1. (0) Indicates the presentation of the tone pip (10-0-10 ms). (A) Marks the start of the ECR, then a first negative peak (B), followed by a first positive peak (C), followed by a second negative peak (D). For a test tone pip of 20 ms and maintaining a distance of 0.60 m between the sound processor microphone and the sound source, *t*_*A*_ = 10 ms, *t*_*B*_ = 12 ms, *t*_*C*_ = 17 ms, *t*_*D*_ = 31 ms, and *t*_wa_ = 50 ms is the analysis window width. These times may vary depending on test tone pip intensity level and CI manufacturer. The amplitudes *B*_amp_, *ECR*_amp_, and *D*_amp_ are the amplitudes of peaks *B*, *C*, and *D*, respectively.

Individual addressing of intracochlear electrodes is achieved by choosing test tone pip frequency equal to the central frequency of the frequency band assigned to the electrode in the device MAP. Thus, changes in ECR amplitude and slope versus tone pip intensity level are obtained individually for each intracochlear electrode.

The amplitude of positive peak *C* is called ECR amplitude, *ECR*_amp_. Negative peaks *B* and *D* mark the start and end of the ECR and time *t*_*A*_ is the time the tone pip takes to travel the distance between the location of the loudspeaker and the position of the patient’s head where the sound processor microphone is located. This separation is necessary to reduce the artifact induced by the loudspeaker on the cables of the EEG recording electrodes.

The ECR wave shape and amplitude depend on the intensity and morphology of the input sound stimulus, the dynamic range of current in the array electrodes, and the auditory nerve responsiveness to electrical current. Given a certain dynamic range of current, by observing the value of the ECR slope and amplitude due to tone pips of frequency equal to that of the central frequency of the electrode under test and variable intensity, it is possible to establish to what extent the operation of the CI, in the electrode’s frequency band, responds to the patient’s needs.

This work reports the results obtained from a population of implanted children, in recording and measuring the ECR generated by presenting in sound field a set of tone pips of variable intensity, 10–90 dB_HL_, and frequency equal to the central frequency of the frequency band assigned to each active intracochlear electrode. The ECR test result allows to establish the ECR growth slope and amplitude versus tone pip intensity level for each of the intracochlear electrodes. These ECR parameters provide insight into the auditory nerve response to tone pips in the everyday use of the CI, and at the same time, allow an objective dynamic range of electrical current adjustment for individual intracochlear electrodes besides the possibility for an objective estimation of hearing thresholds electrode by electrode since the initial CI activation. The ECR does not depend on patient’s cooperation or previous experience in using the device and applies equally to all modern CIs. The potential use of ECR in clinical practice is illustrated by cases, where patient auditory behavior improved after adjusting the dynamic range of current in the electrodes based on ECR test results. The ECR was obtained using equipment designed and constructed for the purpose.

## Results

Total population consisted of 43 implanted pediatric patients, 23 boys and 20 girls, 1.5–6.5 years of age, of whom 26 are* Cochlear* users, 10 are *Medel* users and 7 are *Advanced Bionics* users.

Table 1 shows the mean values and 95% confidence intervals obtained for the three ECR parameters considered for its characterization. There were not statistically significant changes in ECR in relation to manufacturer of CI. Likewise, the *t* student test performed to evaluate changes in ECR by gender showed no significant changes.

**Table 1:** ECR characterization parameters including all cases

ECR parameters	Mean ± SD	95% confidence interval
Threshold amplitude [μV]	4.2 ± 1.8	4.02–4.38
Threshold intensity [dB_HL_]	42 ± 12.7	40.7–43.3
PTA_ECR_ [dB_HL_]	39 ± 15	37–41

Threshold intensity: minimum sound intensity for ECR detection. Threshold amplitude: amplitude ECR at threshold intensity. PTA_ECR_: average threshold intensity in the range of 500–2000 Hz

**Fig. 2:**
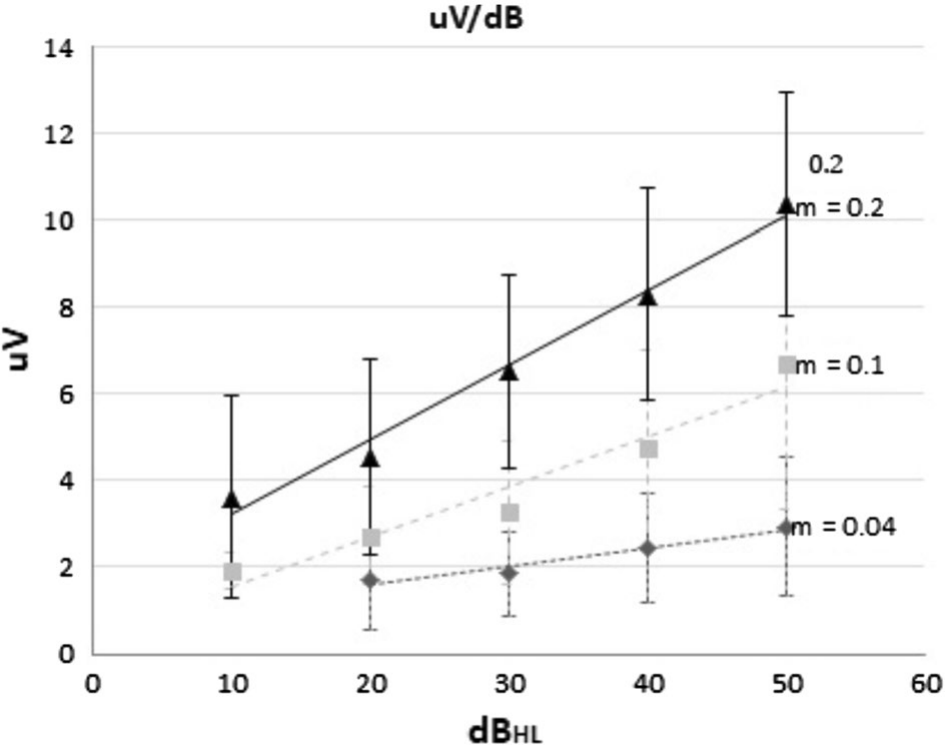
Average ECR amplitude linear regression versus test tone pip intensity level according to patient auditory behavior. (♦) Inconsistent group, (■) acceptable group, and (▲) sensitive group. Notice that sensitive group shows higher sensibility to electric stimulation, *m* = 0.2 µV/dB_HL_, and higher ECR amplitude than inconsistent and acceptable groups

Figure 2 shows the linear regression of average ECR amplitude versus tone pip intensity level, grouping patients based on their auditory behavior. The three groups show the same tendency; however, the sensitive group showed greater ECR amplitude and slope whereas the inconsistent group showed lower values. (♦) Inconsistent group, *y* = 0.04*x* + 0.9; (■) acceptable group, *y* = 0.1*x* + 0.4; and (▲) sensitive group, *y* = 0.2*x* + 1.5. It is noteworthy that, for the same range of intensity, the ECR amplitude of the sensitive group is the highest of the three groups, see Table 2.

**Table 2:** Population grouping according to auditory behavior

Patient auditory behavior	Amplitude [μV] at 50 dB_HL_	Slope [μV/dB_HL_]	Threshold amplitude [μV]	Threshold intensity [dB_H_]	PTA_ECR_ [dB_HL_]
Inconsistent	2.4	0.04	2.9 ± 0.8	54 ± 12	52 ± 14
Acceptable	6.4	0.1	4 ± 1	39 ± 8.7	36 ± 10
Sensitive	10.1	0.2	6.6 ± 2.3	31 ± 7.9	24 ± 9.6

**Fig. 3:**
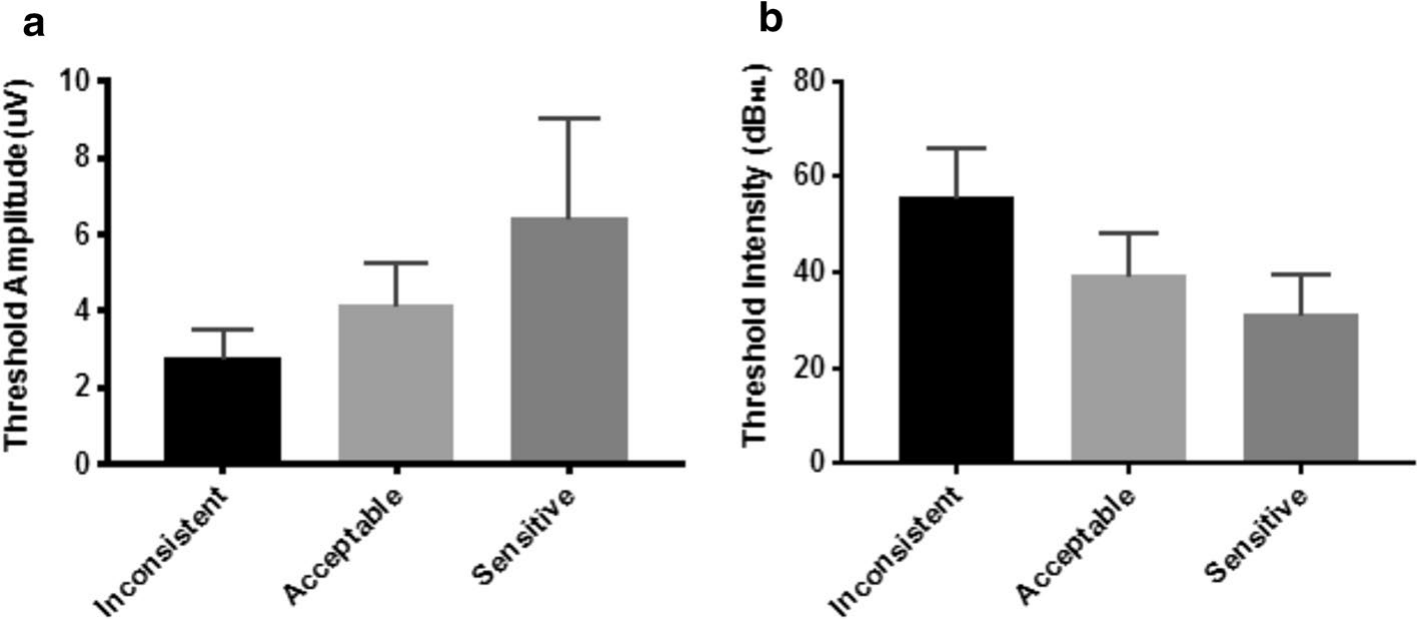
Bar graphs for threshold amplitude (**a**) and threshold intensity (**b**) according to patient auditory behavior

The bar graphs of threshold amplitude and threshold intensity based on patient auditory behavior are shown in Fig. 3. A higher threshold amplitude for the sensitive group compared with the inconsistent group was observed. Significant differences in threshold amplitudes between the three behavior groups was observed (*p* < 0.05). At the same time as the threshold intensity behaves oppositely, i.e., threshold intensity is lower in the sensitive group than in the inconsistent group. There were not significant differences in threshold intensity between acceptable and sensitive groups.

Table 2 shows the slope, threshold amplitude, threshold intensity, and PTA_ECR_ values, in accordance with the grouping of patients by auditory behavior. The slope indicates differences in sensitivity to electrical current between groups. The average PTA_ECR_ values showed significant changes between the three groups (*p* < 0.001). There were not significant differences in PTA_ECR_ values between acceptable and sensitive groups.

### Representative cases

The cases of four implanted patients are shown. The patients’ demographic details are summarized in Table 3. Their ages vary from 1 to 8 years, all of them with prior experience in using the CI, and with different auditory behavior at the time they underwent ECR tests, Table 4. The first one is a case of success observed through ECR. Then, we present the cases of three patients implanted with devices from different manufacturers and with different auditory behavior. The cases are illustrated with ECR readings obtained from intracochlear electrodes located in the basal, medial, and apical regions of the cochlea, before and after readjust of the dynamic range of current guided by the ECR results, Table 5.

**Table 3:** Representative case demographic data

Case	1	2	3	4
Gender	Female	Male	Male	Male
Age	8 years 10 months	3 years 8 months	1 year 4 months	5 years 3 months
Diagnosis	Profound bilateral hearing loss	Profound bilateral hearing loss	Congenital bilateral cortipathy	Congenital bilateral cortipathy
Experience with hearing aids	6 months	7 months	2 months	2 months
Cochlear implant manufacturer	Advanced Bionics	Advanced Bionics	Cochlear	Medel
Implantation age	2 years 3 months	2 years 7 months	1 year	2 years 11 months
Active electrodes	14/16	12/16	22/22	8/12
Experience with cochlear implant	6 years 7 months	1 year 1 month	4 months	2 years 4 months
Sound processor	Harmony	Neptune	Nucleus 5	RONDO
Stimulation strategy	HiRes P Fidelity 120	HiRes Optima-S	ACE	FS4-P
Auditory behavior	Quasi-normal	Inconsistent	Deficient	Sensible

**Table 4:** Patient auditory behavior before and after electrodes dynamic range readjusting

Case	1	2		3		4	
	ECR_1_	ECR_1_	ECR_2_	ECR_1_	ECR_2_	ECR_1_	ECR_2_
CI use time	6 years, 8 months	5 months	7 months	4 months	8 months	2 years, 4 months	2 years, 10 months
Voice development	Clear and quasi-normal	Babbling	Increased babbling	Babbling Words production with pronunciation errors	Increased babbling and words production	Poor acquisition of speech and words pronunciation	Favorable evolution in phrases comprehension and production
Conversation abilities	Sustained conversation without difficulty one meter away from speaker	Not reported	Not reported	Responds to his name at short distance or spoken loudly	Increasing word production	Communicates by shouting	Asks for repetition of words frequently
Sound detection	Can hear whispering	Ling sounds at high volume. Some domestic sounds	Most domestic sounds	Most domestic sounds	Low-intensity domestic sounds	Most domestic sounds	Words, sentences, and Ling sounds
Auditory behavior	Noisy environment tolerance Discomfort to intense sounds	Inconsistent, lacking repetitive-ness, and discomfort to intense sounds	Gradually it became consistent	Not reported	Mild discomfort to shouting and vehicles noise	Removes the CI antenna by himself with intense sounds Facial nerve stimulation without any discomfort	Stopped removing the CI antenna by himself

**Table 5:**
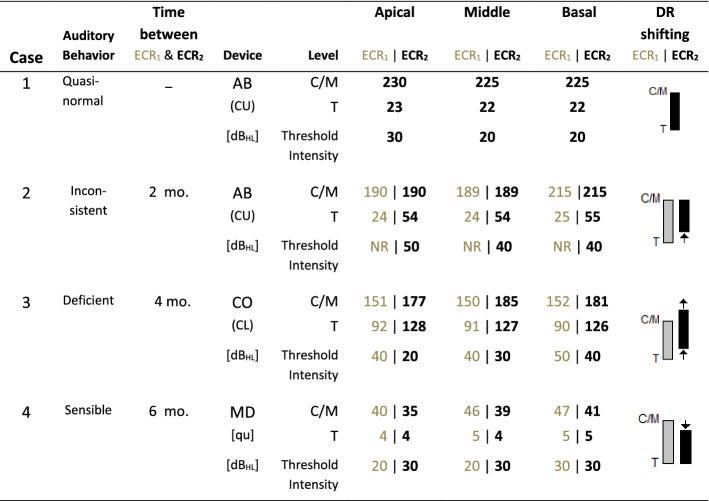
Representative cases

Changes to dynamic range of electrical current of the intracochlear electrodes guided by ECR

### Case 1: Quasi-normal auditory behavior

*Patient:* Female, diagnosed with bilateral profound hearing loss of genetic etiology. Without improvement in hearing with use of bilateral hearing aids. At age 2 years and 3 months, she was implanted with an *Advanced Bionics* cochlear implant with *Harmony* processor on the right side and *HiRes P* stimulation strategy with *Fidelity 120*. Of 16 electrodes in the array, 2 were inactive due to incomplete insertion.

*Clinical impression:* Voice clear and fluid, practically normal, good pronunciation and able to converse without difficulty at a distance of 1 m from her interlocutor. Attends a regular school with good academic performance. She does not report discomfort in moderately noisy environments, although intense sounds annoy her.

**Fig. 4:**
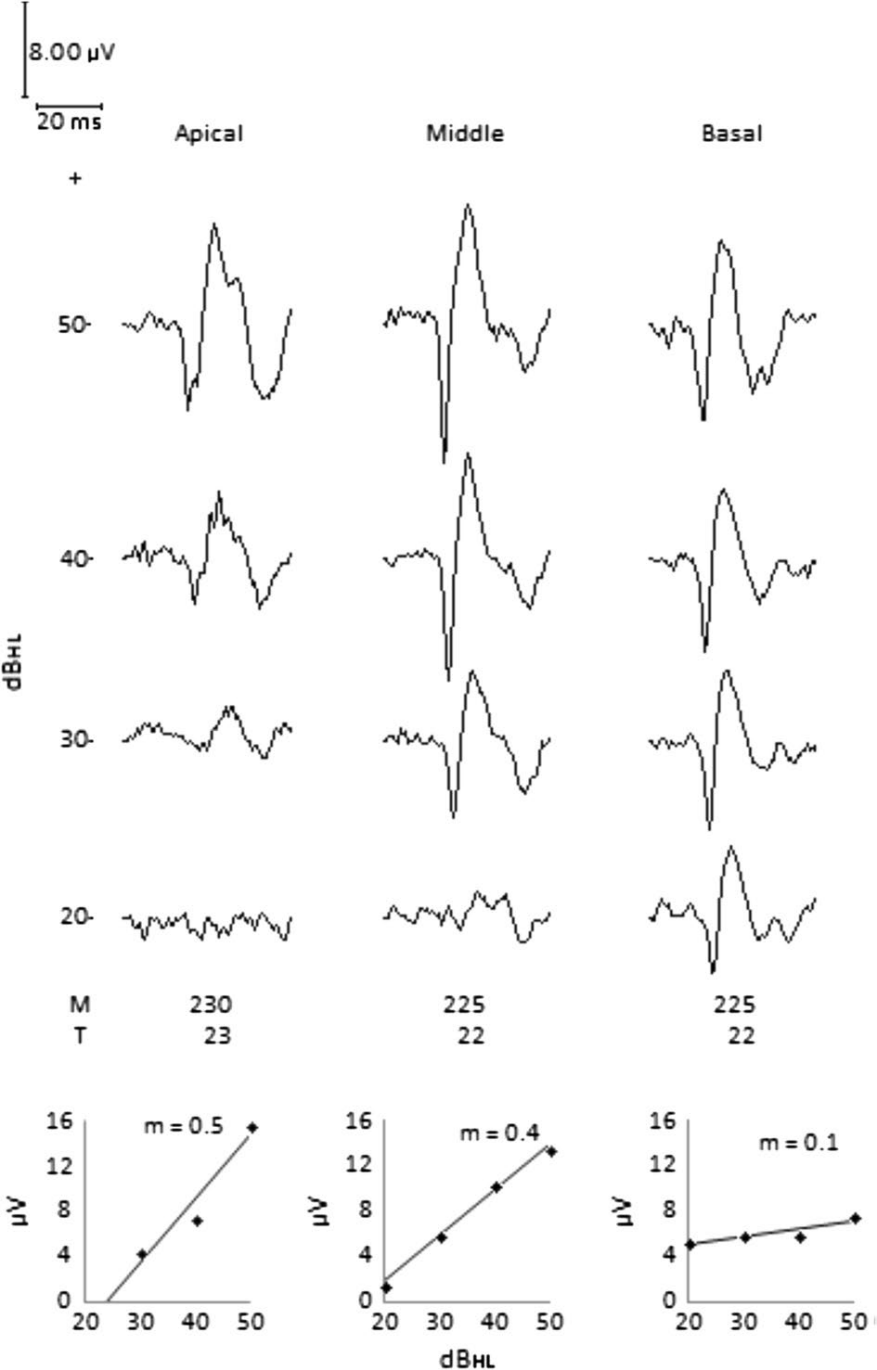
Case 1. Female user with quasi-normal auditory behavior. All electrodes show ECR morphology as described in Fig. 1 and ECR amplitude growing as test tone pip intensity level increases. For the same test tone pip intensity range and similar current dynamic range, the apical electrode shows higher growing slope, 0.5 µV/dB_HL_, than basal electrode, 0.1 µV/dB_HL_. Threshold intensity of 30 dB_HL_ for apical electrode, and 20 dB_HL_ for middle and basal electrodes

*ECR test:* The test was performed at 8 years and 10 months of age, after using the CI for a period of 6 years and 8 months. No significant ECR morphological differences between electrodes were observed, see Fig. 4. The ECR amplitude increases as the intensity of the test tone pip increases. Threshold intensity of 30 dB_HL_ in the apical electrode and 20 dB_HL_ for the medial and basal electrodes. Even when the dynamic range of electrical current of the three electrodes is practically the same, differences in the behavior of the growth slope and ECR amplitude were observed. Based on the ECR slope value, sensitivity to electrical stimulation falls from the apex to the base; however, the amplitude shows opposite behavior.

### Case 2: inconsistent auditory behavior

*Patient*: Male, diagnosed at 2 years and 7 months of age with bilateral profound hearing loss. No improvement of hearing with use of bilateral hearing aids for 7 months prior to implant surgery to place an *Advanced Bionics* cochlear implant with *Neptune* processor on the right side*,* using a *HiResm Optima-S* stimulation strategy. Of 16 electrodes in the array, four were inactive due to incomplete insertion.

*Clinical impression*: When the ECR_1_ test was performed, the patient stammered, detected Ling sounds when speaking aloud, the acoustic alarm of the microwave oven, the telephone ringing, a dog barking, the sound of the blender, and people knocking on the door. However, the patient’s response to sound is inconsistent, he does not repeat the sounds indicated and shows discomfort with intense sounds. The *T* level of the electrodes was raised and 2 months later, ECR_2_ was performed, where the patient’s auditory behavior gradually changed to consistent.

**Fig. 5:**
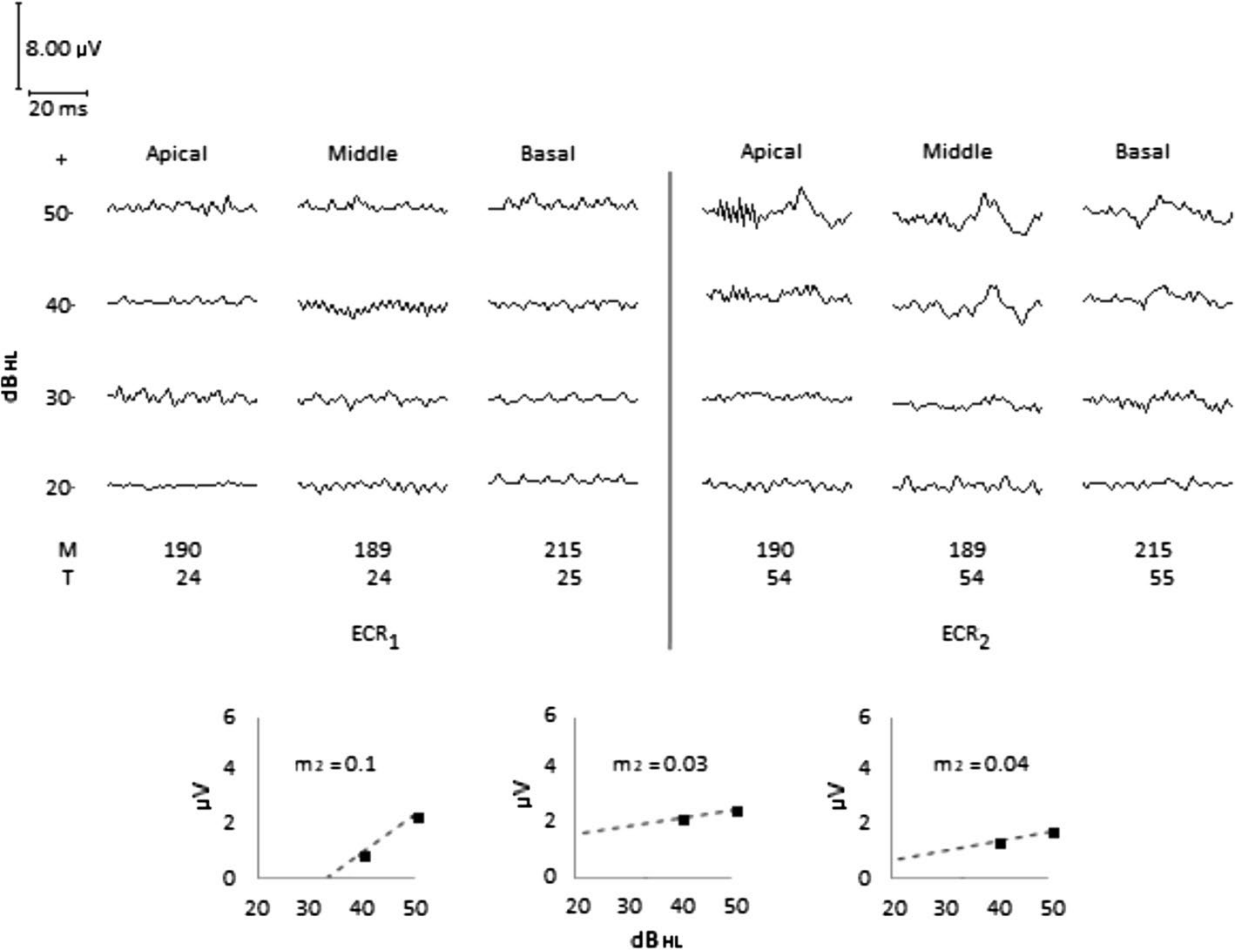
Case 2. Inconsistent auditory behavior. The ECR_1_ test result shows no recognized electrical activity for any electrode for a tone pip intensity level up to 50 dB_HL_. In ECR_2_, after 30 units increasing of the T level for all electrodes ECR was detected. Threshold intensity of 50 dB_HL_ for apical electrode and 40 dB_HL_ for middle and basal electrodes. However, ECR_2_ still shows low sensibility to electric current for all electrodes that reflects on low ECR amplitude compared to Case 1

*ECR test*: Fig. 5 shows the ECR wave shapes. After 5 months using the CI, ECR_1_ was performed. ECR morphology was not detected in any of the electrodes for intensities from 20 to 50 dB_HL_. Even when the test tone pip intensity reached 90 dB_HL_—wave shape not illustrated—without detecting ECR, the patient’s physiological sleep state was not altered. Considering the patient’s auditory behavior and ECR_1_ test result, it was decided to gradually raise the *T* level of the electrodes by 30 units, keeping *M* levels unchanged. Two months later, ECR_2_ was performed, finding threshold intensities of 50 dB_HL_ for the apical electrode and 40 dB_HL_ for the medial and basal electrodes. However, the value of growth slopes indicates low electrode sensitivity, which is reflected in low amplitudes compared with case 1. The initial artifact observed in the wave shapes from the apical electrode at intensities of 40 and 50 dB_HL_ in ECR_2_ are due to the electrical artifact from the loudspeaker on the cables of the recording electrodes.

### Case 3: deficient auditory behavior

*Patient*: Male, 1 year 4 months of age. Diagnosis of congenital bilateral cortipathy. Use of bilateral hearing aids for 2 months prior to implant surgery without hearing improvement. *Cochlear CI422* cochlear implant with *Nucleus 5* processor placed on the left side with stimulation ACE strategy. Full insertion of the electrode array.

*Clinical impression*: When ECR_1_ was performed the patient stammered, paid attention when called by name at close distance or shouting, detected the majority of domestic sounds and produced words with errors in pronunciation. When ECR_2_ was performed, we observed more stammering, detection of low-intensity domestic sounds, higher production of word and better performance in voice therapy. However, the patient reported some discomfort from shouting and noisy vehicles.

**Fig. 6:**
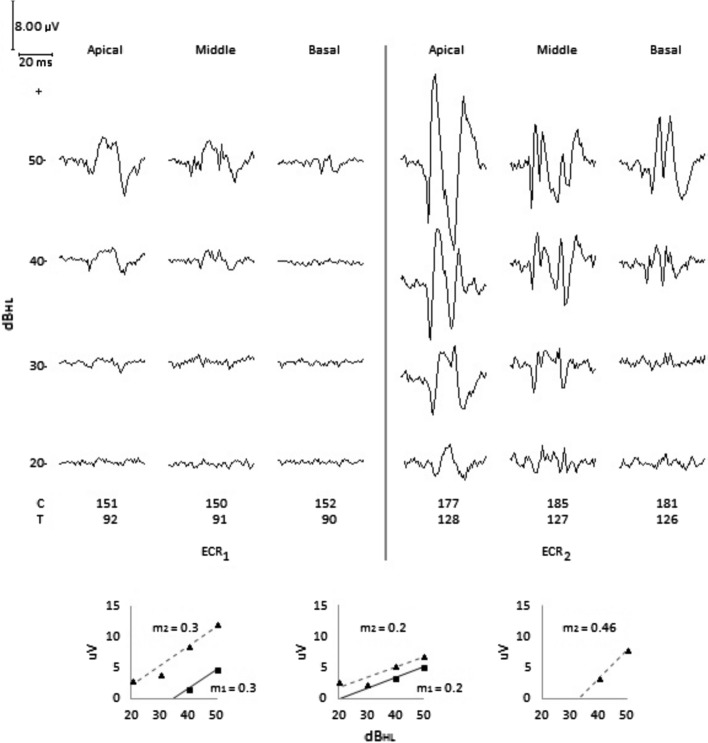
Case 3. Deficient auditory behavior. ECR_1_ test result shows low ECR amplitude for all electrodes, with threshold intensity of 40 dB_HL_ for apical and middle electrodes, and 50 dB_HL_ for basal electrode. In ECR_2_ after increasing *T* and *C* level for all electrodes, the threshold intensity changes to 20, 30 years 40 dB_HL_ for apical, middle and basal electrodes, respectively, and at the same time there is a general ECR amplitude increasing for all electrodes

*ECR test*: ECR_1_ was performed after 4 months using the CI, see Fig. 6. In the apical and medial electrodes, the threshold intensity was 40 dB_HL_, and in the basal electrode, 50 dB_HL_. Due to the low ECR amplitude observed in all the electrodes, it was decided to shift the dynamic range of electrical current of them all toward higher values raising the *T* level by 36 units and the *C* level by 26, 35, and 29 units in the apical, medial, and basal electrodes, respectively. Four months later, ECR_2_ was performed, finding threshold intensities of 20, 30, and 40 dB_HL_ for the apical, medial, and basal electrodes, respectively. A general increase in ECR amplitude proportional to the increase in test tone pip intensity was observed, which was greatest in the apical electrode. The linear regression straight line of the medial and basal electrodes shifted toward higher values, preserving the same slope, i.e., the increase of electrical stimulation led to higher ECR amplitudes, without affecting electrode sensitivity. On the other hand, a threshold intensity of 40, 30 and 20 dB_HL_ for basal, middle and apical electrodes, was observed. As in case 1, sensitivity was greatest for the apical electrode. The ECR morphological differences between these basal and apical electrodes are noteworthy.

### Case 4: sensitive auditory behavior

*Patient*: Male, 5 years 3 months of age. At 2 years and 9 months of age, he was diagnosed with congenital bilateral cortipathy. Bilateral hearing aids were used for 2 months prior to implant surgery without hearing improvement. At 2 years and 11 months of age, a *Medel* cochlear implant with *RONDO* processor was placed on the right side with FS4-P stimulation strategy. Of 12 electrodes in the array, 4 were inactive due to incomplete insertion.

*Clinical impression*: The patient pays attention to a majority of domestic sounds, he removes the CI antenna himself when he is in the presence of intense sounds, he communicates in shouts, presents scant progress in production of words and development of voice. Seeking to improve his perception of sound, the *T* and *M* levels of all the electrodes were raised successively, discontinuing the action on observing stimulation of the facial nerve. In addition, the pulse width was increased without achieving any improvement. Despite his age, the patient lacks the conditioning necessary to achieve reliable audiometry. After performing ECR_1_, the *M* level of the electrodes was lowered. Six months later, when ECR_2_ was performed, an improvement in his auditory behavior was observed. The patient no longer removed the CI antenna himself in the presence of intense sounds and often asks others to repeat words he does not understand. The speech therapist reports improvement in detection of Ling sounds and in identification and comprehension of words and phrases.

**Fig. 7:**
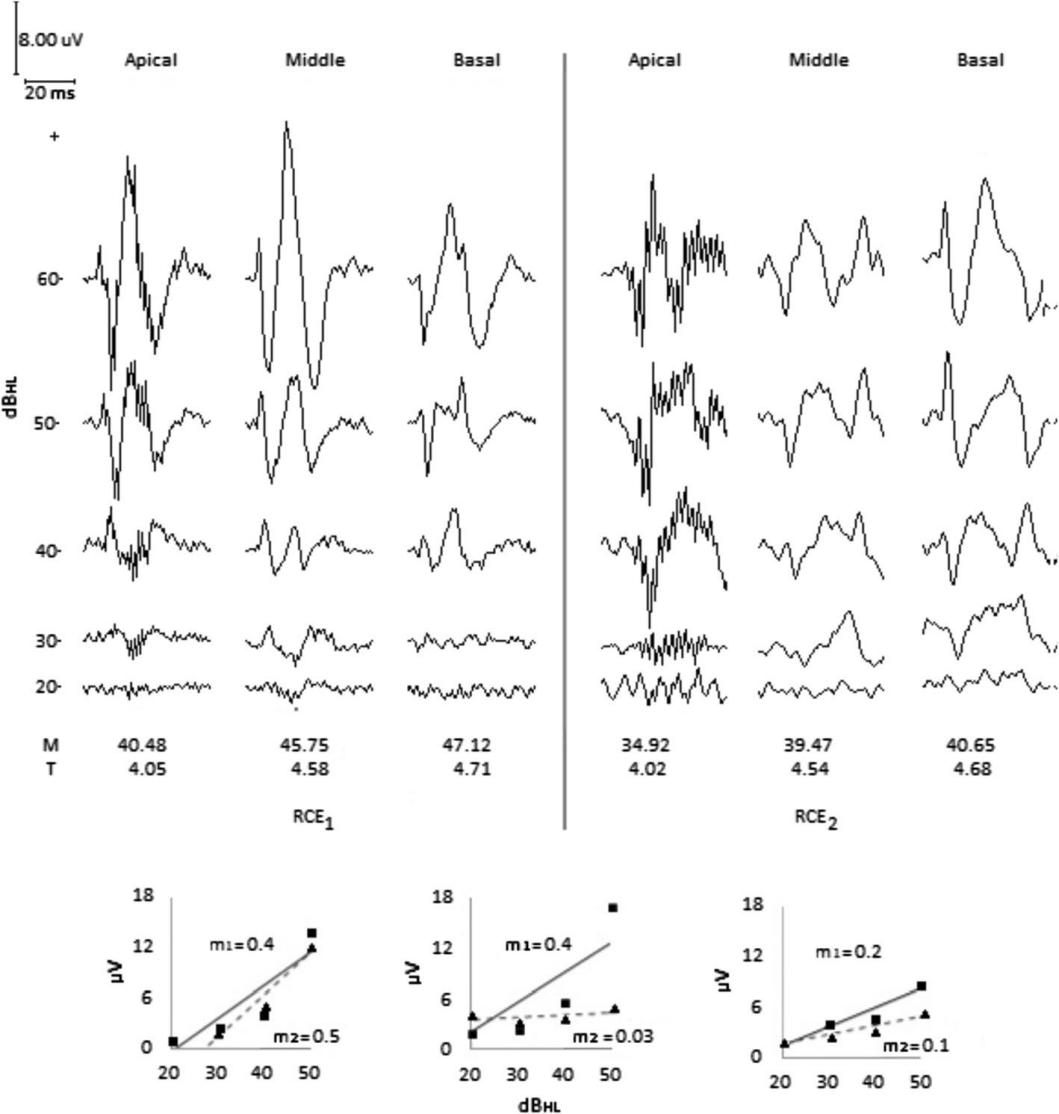
Case 4. Sensible auditory behavior. ECR_1_ test result shows a threshold intensity of 20 dB_HL_ for apical and middle electrodes, and 30 dB_HL_ for basal electrode with slope and amplitude ECR higher than previous cases for all electrodes. After decreasing *M* level and keeping *T* level nearly unchanged for all electrodes, ECR_2_ test result shows a threshold intensity of 20 dB_HL_ and a significant ECR slope and amplitude reduction for all electrodes. Apical electrode initial artifact is typical for this device brand

*ECR test*: In ECR_1_, Fig. 7, a threshold intensity of 20 dB_HL_ for the apical and medial electrodes, and 30 dB_HL_ for the basal electrode was observed. However, the rapid growth of ECR amplitude as tone pip intensity grows is noteworthy, particularly for the apical and medial electrodes. Based on this result and taking into account the patient’s auditory behavior, it was decided to lower the M level of the electrodes by 6 units, without significantly changing the *T* level. ECR_2_ was performed 6 months later, observing ECR morphologies similar to those observed in ECR_1_, although of lower amplitude. A threshold intensity of 30 dB_HL_ was determined for the apical electrode, and 20 dB_HL_ for the medial and basal electrodes, with a lower growth rate of ECR amplitude versus test tone pip intensity level, compared that observed in ECR_1_.

## Discussion

Today, there is no doubt regarding the benefits a cochlear implant provides for the implanted patient, although at the same time individual differences are cited to explain the wide variety of results seen in patients, including among those with the same device model, the same rehabilitation therapy or gender, and similar age and medical histories [18, 19]. Although device programming for different patients may be similar, sensitivity to electrical current among implanted users is unlikely to be the same. In addition, such sensitivity is not constant along the implanted cochlea since it depends on local conditions in the vicinity of the active electrode [9, 10].

Estimating patient’s *T* and *C*/*M* psychophysical levels based on the eCAP threshold may be affected by the change of pulse width and stimulation rate when patient uses the device in everyday mode, in addition to the summating effect which contiguous stimulation pulses has on auditory nerve response [16, 20]. On the other hand, in a group of implanted children [21] found stable eCAP threshold behavior in the first year of CI use, while at the same time the *T* and *C*/*M* psychophysical levels rose. The lack of concordance between the dynamic range of electrical current delivered by electrodes with psychophysical threshold, and comfort levels, along the cochlea leads to clinical scenarios like those described in cases 2, 3, and 4, which are difficult to evaluate and remedy with the battery of clinical tests presently available.

Unlike today’s objective tests, the electrical stimulation used to obtain ECR is the same as that the patient experiences in real CI usage conditions: modulated amplitude trains of pulses presented at a rate of stimulation higher than that used in today’s objective tests.

Choosing a test tone pip frequency equal to the central frequency of the electrode under test, frequency at which the gain of the pass-band filter assigned to the electrode in the sound processor is maximum, combined with 10 ms attack and relaxation slopes result in a test tone pip of 20 ms, thereby attaining sufficient selectivity in frequency to allow for individual testing of intracochlear electrodes, while at the same time avoiding triggering the sound processor automatic gain control, AGC.

The patient’s waking state, in addition to the precautions needed to reduce contamination of the ECR by artifacts, are equal to those used in acquisition of ABR potentials. Special care should be taken to keep the cables of the EEG recording electrodes away from the CI antenna, sound processor, and from the loudspeaker which produces the sound field.

In CI models which use a rate of stimulation higher than that of the other electrodes in the first four apical intracochlear electrodes, the ECR appears masked by high frequency noise, see apical electrode in Fig. 6.

The morphology, amplitude, and latency of the ECR along the implanted cochlea are determined by the asynchronous triggering of auditory fibers based on the intensity and changes in amplitude of the test tone pip. The occurrence and amplitude of such triggering depends on the population density and health status of fibers located along the pathway the electrical current follows between the active and reference electrodes. The time delay seen between the presentation of the stimulus, *t* = 0, and the appearance of ECR, *t* = *t*_*A*_, Fig. 1, comprises the time the test tone pip takes to travel from the loudspeaker to the sound processor microphone, plus the time delay imputable to the device’s response time, which differs among devices from different manufacturers.

The growth of ECR amplitude versus tone pip intensity can be explained considering that an increase in input sound intensity level translates into an increase in the magnitude of the electrical current delivered by the CI current source to the pair of electrodes, thereby increasing the magnitude of stimulation on the auditory nerve. In general, the ECR obtained from different intracochlear electrodes has a similar morphology; however, there are marked morphological variations between apical and basal electrodes which are easier to identify in devices with 22 electrodes. Records made to date indicate that the quality of ECR morphology tends to be better in apical than in basal electrodes, which coincides with better patient perception of low tones. This suggests the probable use of ECR to determine the quality of hearing perceived by the patient in each of the intracochlear electrodes.

In case 1, the patient’s quasi-normal auditory behavior corresponds, in this case, to a practically equal dynamic range in the three electrodes, Fig. 4, observing well-defined wave shapes, low artifact levels, and different ECR growth slopes and threshold intensity among electrodes. It is noteworthy that the threshold intensity of 20 dB_HL_ for the basal electrode and 30 dB_HL_ for the medial and apical electrodes correspond to normal hearing thresholds. It contrasts with the inconsistent behavior of the patient in case 2, for whom ECR_1_ did not detect a response in any electrode for a stimulation intensity level of up to 50 dB_HL_, Fig. 5. These results, combined with auditory behavior, suggested low electrical stimulation. In ECR_2_, performed after raising the *T* level of the electrodes by 30 units, the threshold intensity was 50 dB_HL_ for the apical electrode and 40 dB_HL_ for the medial and basal electrodes, coinciding with an improvement in the patient’s auditory behavior.

In case 3, even though the patient responds to everyday sound, his auditory behavior is unsatisfactory. With a similar dynamic range between electrodes, ECR_1_ shows a threshold intensity of 40 dB_HL_ in the apical and medial electrodes, and no response in the basal electrode. The patient’s auditory behavior combined with the results from ECR_1_ suggested a need to readjust the current dynamic range of the electrodes. Shifting the dynamic range of current toward higher values by raising the *T* level equally and differentiated from the *C* level, results in a threshold intensity of 20 dB_HL_ for the apical and medial electrodes and 40 dB_HL_ for the basal electrode, as shown in ECR_2_, thereby obtaining better auditory behavior, Fig. 6.

The ECR_1_ from case 4 shows ECR amplitudes greater than those seen in the previous cases, particularly in the medial electrode. Such information, combined with the patient’s auditory behavior, raised speculation about excessive stimulation, which was confirmed with differentiated reductions in the *C* level of the electrodes, as shown in ECR_2_, Fig. 7, where the ECR amplitudes are lower than in ECR_1_ but morphologies are preserved, observing improved sound discrimination and less discomfort when exposed to intense sounds in the patient. The findings reported thus far show that determining the dynamic range of current to the electrodes, guided by the ECR result, helped improve auditory behavior in these patients.

The representative cases presented here show that ECR measurement can be used to quantify changes in loudness experienced by the patient due to changes of input sound intensity level, thereby obtaining an auxiliary index useful in determining the dynamic range of stimulation current to electrodes in the array. Systematic use of this methodology may well lead to customized programming of implants for individual patients based on criteria of hearing other than those currently used, related to electrical stimulation.

The above shows the potential clinical use of ECR, suggesting in turn, the advisability of constructing a knowledge base on ECR behavior in different clinical scenarios, which would help to predict the probable development of a patient’s hearing. An immediate consequence of this is to prevent cases such as lower or over electrical stimulation and diminish the time necessary to achieve a proper dynamic range of the electrical current for each electrode in the electrode array, with the importance it entails for an opportune patient rehabilitation.

## Conclusions

The ECR approach to assessing hearing in an implanted patient, as the result of electrical stimulation provided by a cochlear implant, is complementary to that of existing objective tests. Taking the response of the patient’s auditory nerve to electrical stimulation, information collected during or after surgical implantation of the device, as given and having established the dynamic range of electrical current in the intracochlear electrodes, ECR provides information on auditory nerve response to electrical stimulation corresponding to tone pips of variable intensity and known frequencies, in everyday use of the cochlear implant. The correlation of such information with the patient’s auditory behavior helps to confirm or reconsider the dynamic range setting of electrical current in intracochlear electrodes.

On the other hand, the relationship observed between threshold intensity for ECR detection in an electrode and the patient’s auditory threshold therein suggests the possibility of identifying a new resource to estimate patient auditory thresholds as soon as the device is activated for the first time.

Since ECR is a non-invasive and inoffensive test for both the patient and the cochlear implant, the results of which do not depend on the patient’s cooperation or prior experience in using the device, and is applied equally regardless of the device manufacturer, its use includes both pediatric and adult patients. Finally, because observing the ECR assumes that the device is working on properly, when the test is performed, it is possible to detect several malfunction problems in the device.

## Methodology

### ECR system for stimulation, recording, and display

**Fig. 8:**
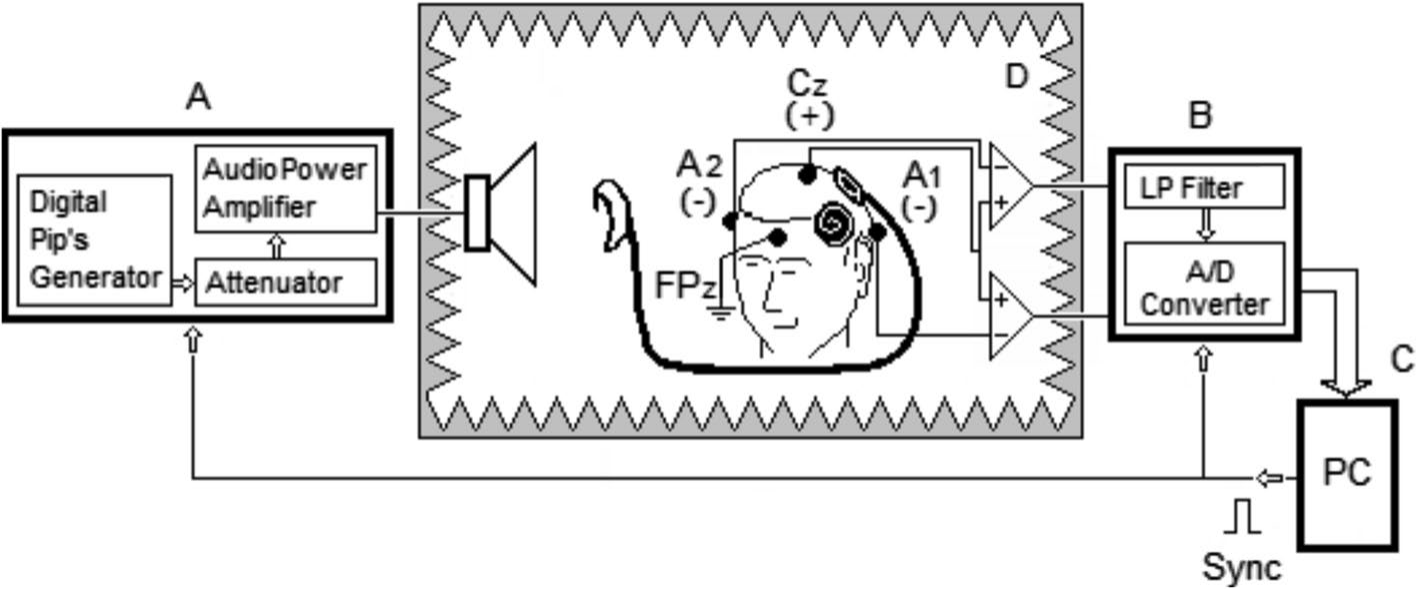
Experimental setup and block diagram of the system for ECR acquisition and recording. Patient asleep inside the audiometric test booth (D) and using the device in everyday operating mode. Four scalp EEG electrodes for two differential recording channels. (A) Stimulation module. Consist of a digital programmable tone pip’s generator, digital attenuator, audio power amplifier and loudspeaker. (B) Acquisition module. Consist of two EEG amplifiers, 30–500 Hz wide band, 0.1–300 Hz LPF, 12 dB/Octave and A/D converter. (C) Personal computer

The system consists of stimulation and acquisition modules, controlled by a personal computer, Fig. 8.

(A) *Stimulation module*: Includes a programmable digital pip generator, digital attenuator, audio power amplifier, and loudspeaker. It generates *m* × *n* pips, where *m *is the number of active intracochlear electrodes and *n* is the number of EEG epochs per intracochlear electrode. Random presentation of tone pips in sound field, from 10 to 90 dB_HL_.

(B) *Acquisition module*: Consists of two EEG acquisition channels with gain of 10^4^, CMRR 60 dB, bandwidth 30–500 Hz, low-pass filter of 0.1–300 Hz with cutoff slope of 12 dB/Octave and artifact rejection window ± 10 µV. 10-bit A/D converter with 0.007 µV/LSB. Electrode–skin impedance meter with graphic interface. Electrical safety tests performed with *Medtester 5000c Biomedical Electrical Safety Analyzer* in accordance with IEC 60601-1 and NOM-137-SSA1 [22, 23].

(C) *Personal computer*: To run the software which generates and controls the presentation of tone pips, and supervise the acquisition and storage of EEG epochs from each electrode. It generates a TTL synchrony signal of 1–50 Hz to synchronize the presentation of tone pips with acquisition of EEG epochs. When the test is conducted, it allows the operator to view the input EEG and the development of averaging of EEG epochs from each intracochlear electrode online. In addition, it stores relevant patient personal data and medical information, as well as the technical features and programming MAP of the CI.

(D) *Test enclosure*: Audiometric chamber measuring 2.5 m long × 2.5 m wide and 1.80 m high. Calibration of the sound field in the chamber in accordance with ANSI S3.6 [24]—frequencies not mentioned in the standard were calibrated using a polynomial characterization of the ratio stimulus sound intensity vs. loudspeaker output voltage—following the substitution method specified in ISO 389-7 [25], for a distance of 0.60 m between the subject’s head and the audio power amplifier loudspeaker. Noise attenuation between the interior and exterior of the chamber was on average above 70 dB_SPL_ in the interval of 100–8 kHz. The calibration equipment used consisted of a B&K 2235 sonometer, B&K 1625 one-third octave filter, and B&K 4230 microphone calibrator.

### Population

Implanted children from three different hospitals, all with diagnosis of bilateral profound hearing loss of neurosensorial origin, prelingual, auditory deprivation period variable, use of hearing aids for at least 2 months prior to implant surgery, without reliable audiometry and initial CI fitting following a behavioral method, with initial dynamic range of electrical current in the electrode array based on the eCAP threshold. Parent informed consent was obtained for each child participating in the study.

### ECR test

All tests were performed at Metropolitan Autonomous University, Audiology Laboratory. Tests were conducted in the morning, with the patient kept awake 6 h the night before the test. Four, 10-mm diameter gold electrodes were placed in positions A1 (−), A2 (−), Cz (+), and FPz (GND), with impedance of less than 5 kΩ [26] between electrodes. Then, the patient was placed in the audiometric chamber asleep and lying on a reclinable sofa. The CI, with batteries in good condition, working properly, and selecting the stimulation program or MAP prescribed by the audiologist, was fitted to the patient when asleep, taking care not to obstruct the sound processor microphone. At all times, the child’s parent was in charge of handling the device. During the test, the patient remained asleep for 45–90 min, depending on the number of active electrodes in the device, with the light in the audiometric chamber off and accompanied by one of their parents. In some cases, the duration of the test increased due to movements by the patient which required temporary interruption of the test or when the child awoke to then continue sleeping. To monitor the patient’s condition and detect events which might interfere with the proper execution of the test, the interior of the audiometric chamber was monitored at all times through a night vision CCTV system. Test tone pips were presented randomly with increasing intensity, from 10 to 90 dB_HL_ in 10 dB_HL_ increments for each test frequency. In synchrony with the presentation of tone pips, 50 ms EEG epochs were acquired, of which the average in groups of 100 was used to obtain the corresponding electrode ECR for each test intensity. Even when the artifact due to CI operation was present during the test because it was not in synchrony with the presentation of stimuli, it could be canceled by averaging EEG epochs to obtain the ECR.

### Data analysis

Patients were grouped by manufacturer of CI, gender, and auditory behavior: inconsistent, acceptable, and sensitive, based on their parents’ opinion. Inconsistent: erratic detection and identification of sounds and voice, and scant verbal communication. Acceptable: good detection and identification of sounds and voice, and development of verbal communication. Sensitive: discomfort with intense sounds, poor discrimination of sounds, and incipient speech development. Using measurements of ECR amplitude based on tone pip intensity, some relevant figures were defined for the electrode array. *Threshold intensity*: minimum level of sound intensity for robust detection of ECR; *Threshold amplitude*: ECR amplitude corresponding to the threshold intensity, and *PTA*_*ECR*_: average threshold intensity in the 500–2000 Hz frequency range.

*Statistical analysis*: Levene test for equality of variances was used. A Kruskal–Wallis test was used to evaluate changes in threshold intensity, threshold amplitude, and PTA_ECR_, in relation to patient auditory behavior and manufacturer of CI. A Dunn’s multiple comparison test was used to identify differences between auditory behavior groups. A *t*-Student test for independent samples was used to evaluate such changes relative to gender. The data were analyzed with GraphPad Prism 7 software, considering a value of *p* < 0.05; the results are presented as mean ± SD. Confidence intervals of 95% of the mean are included.

## Data Availability

Not applicable.

## Abbreviations

*B*_amp_: ECR peak *B* amplitude
*C* or MC: Psychophysical auditory comfort level or maximum auditory comfort level
CI: Cochlear implant
*D*_amp_: ECR peak *D* amplitude
eABR: Electrical auditory brainstem response
eCAP: Electrical compound action potential
ECR: Electrical cochlear response
ECR_amp_: ECR peak *C* amplitude
eSRT: Electrically evoked stapedial reflex threshold
PTA_ECR_: Average threshold intensity in the 500–2000 Hz frequency range
*T*: Psychophysical auditory threshold
